# Implementation science issues in understanding, collecting, and using cost estimates: a multi-stakeholder perspective

**DOI:** 10.1186/s13012-021-01143-x

**Published:** 2021-08-03

**Authors:** Andria B. Eisman, Andrew Quanbeck, Mark Bounthavong, Laura Panattoni, Russell E. Glasgow

**Affiliations:** 1 Community Health, Division of Kinesiology, Health and Sport Studies, College of Education, Wayne State University, 2153 Faculty/Administration Building, 656 West Kirby, Detroit, MI 48202 USA; 2 Center for Health and Community Impact (CHCI), Wayne State University, Detroit, MI USA; 3 Department of Family Medicine and Community Health, University of Wisconsin, Madison, WI USA; 4 Veterans Administration Health Economics Resource Center and Center, VA Palo Healthcare System, Menlo Park, CA USA; 5 VA Center for Innovation to Implementation (Ci2i), VA Palo Healthcare System, Menlo Park, CA USA; 6 UCSD Skaggs School of Pharmacy and Pharmaceutical Sciences, San Diego, CA USA; 7 Hutchinson Institute for Cancer Outcomes Research, Fred Hutchinson Cancer Research Center, Seattle, WA USA; 8 Dissemination and Implementation Science Program of ACCORDS (Adult and Child Consortium for Health Outcomes Research and Delivery Science), University of Colorado School of Medicine, Aurora, CO USA

**Keywords:** Implementation, Costs, Stakeholder, Perspective, Decision-making, Context, Dissemination, Health economics, Sustainment

## Abstract

Understanding the resources needed to achieve desired implementation and effectiveness outcomes is essential to implementing and sustaining evidence-based practices (EBPs). Despite this frequent observation, cost and economic measurement and reporting are rare, but becoming more frequent in implementation science, and when present is seldom reported from the perspective of multiple stakeholders (e.g., the organization, supervisory team), including those who will ultimately implement and sustain EBPs.

Incorporating a multi-level framework is useful for understanding and integrating the perspectives and priorities of the diverse set of stakeholders involved in implementation. Stakeholders across levels, from patients to delivery staff to health systems, experience different economic impacts (costs, benefit, and value) related to EBP implementation and have different perspectives on these issues. Economic theory can aid in understanding multi-level perspectives and approaches to addressing potential conflict across perspectives.

This paper provides examples of key cost components especially important to different types of stakeholders. It provides specific guidance and recommendations for cost assessment activities that address the concerns of various stakeholder groups, identifies areas of agreement and conflict in priorities, and outlines theoretically informed approaches to understanding conflicts among stakeholder groups and processes to address them. Involving stakeholders throughout the implementation process and presenting economic information in ways that are clear and meaningful to different stakeholder groups can aid in maximizing benefits within the context of limited resources. We posit that such approaches are vital to advancing economic evaluation in implementation science. Finally, we identify directions for future research and application.

Considering a range of stakeholders is critical to informing economic evaluation that will support appropriate decisions about resource allocation across contexts to inform decisions about successful adoption, implementation, and sustainment. Not all perspectives need to be addressed in a given project but identifying and understanding perspectives of multiple groups of key stakeholders including patients and direct implementation staff not often explicitly considered in traditional economic evaluation are needed in implementation research.

Contribution to the literature

- Assessment of cost and implementation time required to implement EBPs is centrally relevant to implementation science.

- Costs vary from the perspective of different stakeholder types (e.g., society, healthcare organization, practitioner, delivery staff, participant).

- Key cost-related issues from the perspectives of different stakeholders are summarized, including those such as patients and delivery staff that are often not explicitly identified and included in economic evaluation.

- This article summarizes the application of economic theory to inform multi-level stakeholder perspectives.

- Recommendations are made for future pragmatic research, application of cost assessment, and its use for decision-making with different stakeholder types.

## Introduction

An integral component of assessing value for implementation efforts is capturing the costs and benefits of implementing evidence-based practices (EBPs) relevant to various groups of stakeholders [1]. Estimating costs and conducting comparative economic analyses, such as cost-effectiveness analysis and budget impact analysis, can be undertaken from several different perspectives. The perspectives taken in an economic evaluation depend on many things, including the primary objective, the clinical or community context, and relevant decision-makers and stakeholders who will utilize the information [2]. Many potential costs exist related to implementing EBPs, but the study perspective is the key determinant of which costs will be relevant to the analysis [3]. Although the traditional “reference case” perspectives for estimating and evaluating costs and benefits include the societal and healthcare sector perspectives [2], the Second Panel on Cost-Effectiveness in Health and Medicine also recommends conducting economic evaluations from the narrower perspectives related to the particular interests of key stakeholders [4]. But the perspectives included also reflect value judgments within an economic evaluation; that is, which perspective or perspectives are most important or relevant [3]. When conducting an economic evaluation in implementation science, then, how do we decide which perspective or perspectives to include? The pragmatic answer is the perspectives of the stakeholder and decision-makers who will be informed by the analysis should be prioritized [3].

Local economic considerations are a critical factor influencing whether or not individuals and organizations, versus larger healthcare systems and society, adopt and sustain EBPs [5]. The burden of implementation costs is often borne by local organizations and decision-makers; information from this perspective is key to informing resource allocation that can impact how well EBPs are adopted and sustained [6]. Healthcare and other service areas (e.g., education) are plagued by a persistent, largely unexplained failure to launch EBPs—even when there is strong empirical support for the value of the intervention. And, even if adopted, they are frequently not sustained [7, 8]. This inconsistency may be, in part, because the different incentive structures facing key stakeholders are not typically considered in the economic evaluations, including within implementation science.

Implementation costs and effects accrue at different levels and are variable across different parties. Costs across these different levels have a notable impact on implementation success and sustainment. A recent systematic review of implementation costing methods by Bowser et al. reflects this reality; researchers found a wide range of costing perspectives represented in implementation research, including organizational, facility, provider, and societal perspectives [9]. Yet, traditional economic evaluation does not always account for costs across these levels. Implementation failure is due often to organizations not being prepared to invest in and support effective EBP implementation or not understanding a priori what costs across what levels, given their organizational structure, they will accrue. Organizations adopting EBPs need to know what it will cost THEM—in their setting, given the resources available, with consideration of staff capacity, workflows, and patient/participant population [6, 10]. Implementation involves different types of people with diverse values and perspectives who are collectively deciding whether or not to adopt and ultimately sustain the EBPs that researchers have developed. We have a critical need to focus on the perspectives most relevant to real-world decision-making and especially implementation and sustainment, such as the patient, clinician, delivery staff, organizational, or payer perspectives [11]. In particular, the societal perspective typically used for cost-effectiveness analysis in healthcare aggregates diverse stakeholder perspectives into a single, global perspective, but the accrual of value is often unevenly distributed across stakeholder groups. Each of these stakeholder groups, however, has different perspectives and different values on which they base economic decisions [12].

The purposes of this paper are to (1) apply a multi-level framework to identify key types of stakeholders in implementation efforts and what cost information (broadly defined) they need for decision making; (2) discuss how we consider simultaneously multiple perspectives and options for applying economic theory to advance coordination across perspectives; (3) address the role of stakeholder partnerships and engagement in determining relevant implementation cost data, pragmatic costing tools, and reporting guides to facilitate this; and (4) present examples of multi-level cost application and provide recommendations and future directions in the field.

## Multi-level framework for stakeholder groups

Incorporating a multi-level framework is useful for integrating the perspectives and priorities of the diverse set of stakeholders involved in implementation research. Ferlie and Shortell’s [13] multi-level model of system change offers a guide to consider various economic perspectives in the implementation of EBPs (see Table 1). Our adapted model includes five levels: (1) the policy and economic environment (e.g., regulatory, financial, payment regimes, and markets), under which organizations, delivery teams, and individual patients operate (e.g., patient, student, family); (2) the organization (e.g., hospital, clinic, school, etc.) that supports the development and work of teams by providing infrastructure and resources; (3) the management team: supervisory staff who are responsible for staffing and other resource allocation decisions; (4) the provider team: professional care providers or the front line staff who implement the EBP; and (5) the individual intervention participant (e.g., patient, student, family).

**Table 1:** Key cost considerations mapped to perspectives, priorities, and stage of implementation for different types of stakeholders

Stakeholder perspective	Key priorities	Example pre-implementation costs	Example implementation costs	Example sustainability costs
Policy and economic environment	Incentivize most cost-effective actions; maximize QALYS^a^	Current market (e.g., capacity, needs assessment); gaps in quality of care	Direct costs (e.g., labor, supplies); factor prices, (fidelity, production scale, distribution, sunk costs); downstream costs [14]	Can provide less reimbursement; consider other incentives; maximize/optimize staff resources
Organization	Stay within budget; align with the mission	Capital expenses; costs of promotion and recruitment; health information technology (e.g., dashboard development)	Time until recoup investments; return on investment (ROI) [15]	Costs to maintain quality service (e.g., labor; technology support/maintenance)
Management team: supervisory staff	Effective allocation of staff; efficient workflow, maximizing outcomes given budget constraints	Training costs; infrastructure development	Costs to produce quality results; documenting and logging time and effort	Retraining costs; cost audit and feedback; budget constraints
Provider team: front line delivery staff	Improve workflow; competing demands; relative benefit of adopting new EBP (time and productivity/outcomes)	Opportunity costs for training, logistics, and preparation for EBP adoption	Time required (e.g., documenting and logging time and effort)	Opportunity costs for ongoing support activities, training; incentives
Individual participant/patient	Improved health outcomes; competing demand; satisfaction; reduce out of pocket expenses	Travel and time costs; opportunity costs; information costs	Time required on regular basis;	Adherence costs; incentives

^a^*QALYs* quality-adjusted life years

Table 1 provides a summary of key cost considerations across different perspectives and implementation phases with examples of costs and priorities. Implementation resource requirements change over time, that is, across implementation phases and vary across stakeholder groups. We offer the following school-based example related to Table 1, focusing on pre-implementation costs. A state department of education notes an increase in reports of mental health issues among students. As a result, the department leadership (policy and economic environment; outer context) mandates that districts across the state offer social and emotional learning (SEL) curricula. Such mandates can be unfunded, underfunded, or funded [16]. In this case, the decision-makers are responding to a current gap in “care” but do not have specific funds to allocate to this effort so they decide to reallocate a limited amount of funding from after-school programming to pay for the interventions only (i.e., not their implementation). Their key priority is to incentivize districts to engage in SEL instruction to reduce the statewide economic and social burden of youth mental health issues. The organizations, in this case, the school districts, must consider how to incorporate this new mandate and stay within the district budget. They consider the costs not just of the intervention, which is covered by the mandate, but the costs associated with implementation including identifying suitable curricular options, ensuring the options are compatible with current district technology and other mental health initiatives, and costs of training school staff in the new EBP. They must also consider that this mandate will result in reduced funding for their current after-school programming. The management team, in this case, the principals and other school-level leadership, must pick from a set of alternatives in consideration of the schools’ overall workload, competing demands, and consideration of opportunity cost or the (health) benefits lost from other alternatives when one is chosen [2]. In this case, the school may have to divert resources and staff time from other initiatives (e.g., after-school programs, substance use prevention programming) for enhanced SEL instruction [16]. For frontline delivery staff (e.g., teachers), time is the primary resource concern when faced with an adoption decision [1]. There will be opportunity costs related to the time needed for training, logistics, and preparation for adopting a new SEL curriculum. For the individual participant (i.e., student), there will be opportunity costs related to the foregone instruction on other health-related issues (e.g., after school time/support, substance use). This example illustrates that the decision to adopt an evidence-based practice must represent a clear and sequential win-win scenario from the perspectives of multiple stakeholder levels; failure at any level can lead to failure in EBP adoption and sustainment. Cooperation and coordination across multiple stakeholders, including those perspectives not routinely considered in economic factors related to implementation (e.g., patients and frontline providers), are required for successful implementation and sustainment.

## Economic theory and multi-level stakeholder perspectives

The purpose of economic evaluation in healthcare is to maximize outcomes related to health that are subject to a set of constraints [17]. This purpose, however, raises important questions about which outcomes will be maximized, what constraints exist, and how this may differ according to various perspectives. Which costs are and benefits are most relevant, however, is inherently dependent on the perspective. Decision-makers may not recognize the impact of their decisions on costs and outcomes from different perspectives because it is not within their scope or their direct concern [17]. When decisions are made without sufficient consideration of their impacts on costs and benefits across different perspectives, they will seldom maximize overall benefits under given resource constraints.

Applying economic theory in the context of implementation research can aid in identifying and understanding multi-level perspectives and approaches to integrating these perspectives. Applying principles of economic theory can also help increase the relevance of implementation cost information used in decision-making across different stakeholder groups to ultimately enhance uptake, implementation, and sustainment of EBPs. We provide an example of applying economic theory to implementation issues using multiple stakeholder perspectives. These examples illustrate important first steps in recognizing potential areas of conflict or unintended economic effects across stakeholder groups and developing plans for resolution. Economic theory can be useful in providing guidance and support around decision-making in the implementation of health services [17], but there are currently few such examples.

### Cooperative game theory

Understanding the distribution of costs and benefits across stakeholders is critical to designing implementation strategies that facilitate economic cooperation among stakeholders. There are numerous examples in implementation science when stakeholders can reduce total joint costs and realize savings by pooling resources and cooperating. Healthcare delivery organizations entering into accountable care organizations entering into bundled payment models must allocate the total costs and savings from episodes of care [18]. Alternatively, organizational managers and frontline providers can allocate time-consuming tasks according to principles that promote collaboration. In these cases, each stakeholder may face different gains and costs related to cooperating and possess different bargaining power. The question becomes how to allocate the gains (benefits and costs) from cooperation to ensure each stakeholder is incentivized to collaborate. While the role of cooperative game theory and specific methods of determining optimal costs and benefits across different stakeholder groups [19] can be useful in informing economic evaluation, the practical application of these theories in implementation science is still developing. These theoretically informed allocation methods, however, have the potential to facilitate economic collaboration across stakeholders to ultimately support sustainable economic and health outcomes that would not be possible when stakeholders act independently.

Theories from related fields such as decision sciences (making optimal choices based on available information; study of cognitive biases) and behavioral economics (concerned about the bounds of rationality for decision-makers) can also aid in understanding multiple stakeholder perspectives when considering costs and benefits of specific courses of action [20, 21]. A full review of relevant economic theoretical constructs, behavioral economics, and decision science approaches and their potential application to multi-stakeholder perspectives in implementation issues is beyond the scope of this paper. Our point is to highlight the potential role theories from other fields can play in considering multi-level stakeholder perspectives to advance the objectives of economic evaluation and to identify this as an important area for future research.

## Stakeholder engagement

Stakeholder engagement is vital for closing the gap between research and practice. The implementation science field increasingly recognizes that participatory approaches are foundational to successful implementation and sustainment, including from an economic perspective [22]. Community engagement can occur along a continuum, from stakeholders as participants in research with little or no active participation to equal partners in engaged across all stages of the research process [22–24]. Methods such as Community-Based Participatory Research (CBPR) can increase the impact of economic evaluation of implementation by increasing both the rigor and relevance of the research; this is accomplished through engaging the stakeholders affected by and making decisions about the allocation of resources [22]. A comprehensive discussion of the wide range of stakeholder engagement methods is beyond the scope of this article, but they vary by factors such as stakeholder group, purpose, budget, time, and staffing [22–24]. There are even resources available to assist in the selection of engagement methods for a given implementation project [25]. And while suitable levels of partner engagement across this continuum may vary, we encourage those embarking on economic evaluation of implementation to employ methods such as CBPR. Such approaches have notable potential to accelerate bridging the gap between research and practice and enhance the likelihood of developing feasible and sustainable policies, programs, and strategies to address identified problems [22].

Understanding cost and benefit implications and perceived value from various stakeholder perspectives, from end-users to delivery staff to the larger system/environment, and what implementation cost information is most beneficial for each of these groups is critical in advancing economic analyses in implementation research. Stakeholder engagement across levels is especially critical when organizations face overwhelming competing demands and scarce resources and/or stakeholders experience disproportionate costs related to implementation, such as opportunity costs for frontline staff engaging in implementation efforts in lieu of billable patient care [9]. Assessing the perspective of different stakeholders has the potential to uncover conflicting priorities on costs and other economic issues. This can create challenges for both research reporting and practical application. To maximize the chances of successful adoption, implementation, and sustainment, it is important to understand the perspectives of all relevant stakeholders for a given implementation effort. When summarizing results for decision-makers in cases where there are discrepancies among stakeholders, we would benefit from presenting results in transparent ways that illustrate the impact of different perspectives. Often this can include sensitivity analyses based on different perspectives and assumptions related to these perspectives. Identifying optimal ways to facilitate decision-making and program/implementation strategy adaptations when perspectives differ is an area for future investigation.

### Low-resource contexts

Multi-level perspectives are especially important when considering implementation efforts in low-resource contexts. For example, if the organizational leadership wishes to implement an EBP to reduce system costs (e.g., a fall prevention program) but the resources required to effectively adopt, implement, and sustain it, such as substantial time from highly paid professional staff (provider team), is not aligned with resource capacity within a setting, the likelihood of implementation success is low. Such misalignment can exacerbate rather than mitigate health disparities [26]. Settings such as community health centers, rural primary care clinics, budget-strapped school systems, inner-city community-based organizations, or low-income countries need to consider the resource constraints of the context when embarking on EBP implementation. Considering cost-related implementation issues in less-resourced contexts, whether they be organizations, communities, or countries, is understudied and requires several additional considerations.

First, while it may still be possible to implement an EBP in a low-resource setting, substantial, and potentially costly, adaptations to the intervention and/or implementation strategy are often needed. Identifying and conducting initial rapid cost estimates of lower-cost implementation strategies for an EBP is vital to addressing equity issues in implementation and sustainment. Conducting this type of rapid cost estimate requires considering the organizational/community capacity and resources from multiple perspectives (e.g., organization, management, and provider teams). Discussion of how to tailor EBPs and implementation strategies to low resource settings and changing context is a separate topic addressed in detail elsewhere [27, 28], but considering the costs and benefits in the face of competing demands and scarce resources are vital in achieving desired outcomes and mitigating health disparities.

Second, both researchers and stakeholders may consider adopting innovative approaches that substantially reduce costs commonly seen in implementation efforts in low- and middle-income countries. In some instances, resource-challenged settings have demonstrated high levels of success in implementation and health outcomes at a dramatically reduced cost. This is sometimes achieved by the use of “task-shifting” in which delivery of an EBP is accomplished by much lower level—and thus lower cost—staff members such as community health workers or peer coaches [29]. Utilizing information from innovative implementation efforts designed to reduce costs, as within lower-income countries, has the potential to guide implementation across settings to maximize health outcomes and minimize costs.

## Multi-level perspectives in cost reporting

While reporting guidelines exist for economic evaluation, including the Consolidated Health Economic Evaluation Reporting Standards (CHEERS), these guides focus primarily on healthcare payer or societal perspectives [30]. While comprehensive, the societal approach aggregates costs across all perspectives and thus can limit understanding of the economic implications of implementation specific to each stakeholder group. Including multiple perspectives, while analytically burdensome, offers the opportunity to evaluate the economic consequences of different implementation decisions from various viewpoints [2]. This expanded consideration of multi-level perspectives offers a bridge to the practical application of economic research in the field of implementation science. A key next step in achieving this objective is designing implementation economic reporting guidelines based on established guides such as CHEERS for practical application in healthcare and other community organizations.

Developing reporting guides can aid in identifying areas of cooperation and conflict and in applying theories from economics, behavioral economics, and decision sciences, to address economic barriers within and across stakeholder groups. This is consistent with conclusions from a recent systematic review whereby researchers recommended standardized guidelines around costing perspectives, instrumentation, and reporting as critical next steps to advance the field [9]. At the individual participant level, for example, the priority may be to improve their health outcomes, satisfaction, or quality of life; in contrast, at the organizational level, the priority is often staying within budget. Such standardized guides can support cost reporting from multiple perspectives. When we prospectively account for multiple perspectives, we can enhance the likelihood of satisfying multiple stakeholders when determining implementation priorities; this will ultimately advance the public health impact and sustainment of evidence-based intervention(s) and related implementation strategies. Yet, it is also important that “analysts undertake ‘costing in context’” even within each analytic perspective; at a particular level, for example, the organization, the priorities may be different; that is, some organizations care about budget, others mission, and others profit [3]. The developing application of decision sciences and economic theory in implementation science, in combination with other developing areas such as mixed methods in economic evaluation of implementation, will aid in developing costing tools most relevant to the context.

Another important consideration is the reporting of uncertainties in cost estimates and cost-effectiveness/benefit analyses. Each cost and benefit input (parameter) includes specific model/structural, and methodological assumptions (e.g., costs remain stable over time) that can influence estimates and, ultimately, conclusions from economic evaluations. Various types of sensitivity analysis can be performed to investigate the influence of these uncertainties on the economic output. For instance, will small changes in an input parameter, such as provider or staff type or time, lead to a big change in cost and/or the relative benefit of adopting an innovation? These sensitivity analyses, from simple (one-way sensitivity analysis) to complex (probabilistic sensitivity analysis), can provide stakeholders with some estimates of the model parameter’s impact on the economic output. For example, increasing the labor cost of front-line staff from the base-case assumption may change an implementation plan from being cost-effective to not cost-effective. An example of sensitivity analysis and the information it can provide to stakeholders is included in our case example. Another option may be to build user-friendly model tools, such as described previously, so that different organizations could easily change some of the input assumptions to fit their specific needs; this is, again supporting the concept of “costing in context” [3]. For example, organization A could include costs related to facilitation and professional learning communities, but organization B would only include facilitation. And they could vary assumptions to fit their conditions (e.g., organization A has labor costs of $25/h, organization B has $20/h labor costs). Such tools could also include expanded outputs, and thus be more instructive for different stakeholders [31]. For example, the model output could show “hours worked” by occupation under various scenarios. You could see under “status quo,” nurses work 40 h/week, but under the “intervention (i.e., implementation strategy condition),” they work 45 h/week. In this way, the organizations would know that they either need to hire more nurses or pay overtime to keep the nurses on board with the intervention implementation. Tools to support standardization of multi-level costing approaches and reporting will also aid in building the business case for implementation science; this will facilitate harmonizing data across implementation studies and provide needed empirical evidence for the costs and benefits of implementation efforts for all stakeholders [9].

This illustration above demonstrates some of the complexities involved in data collection and reporting the results of economic analyses in implementation research. These issues are especially consequential when needing to summarize findings for either non-economist audiences or organizational decision-makers. In our experience, most decision-makers in potential adopting sites are not concerned with either the costs in a randomized research study or detailed economic issues such as marginal costs and economies of scale. Rather, they are concerned with what it would cost them to implement a program in question in their setting—e.g., replication costs. The underlying economic factors such as marginal costs would come into play when calculating and presenting options and potential adaptations (using sensitivity analyses), but decision-makers are often frustrated by many details, qualifications, or jargon from either economics or implementation science. Rather they want to know “what is the bottom line for me in this setting under our conditions.”

## Case example: multi-level perspectives in implementation science

### Frontline providers and organizational perspectives: implementing a fall prevention program

Frontline providers are often the ones who are most impacted daily by the implementation of new evidence-based practices. Taken as a group, front-line providers are generally interested in patient care and safety and the resources required to achieve those goals. For instance, frontline hospital staff involved in a patient safety intervention identified patient safety as a priority; but they cited a lack of proper equipment/supplies and facility issues as major operational barriers that their institutions did not prioritize when implementing a new policy [32]. Additionally, when considering the costs (which are usually predominantly dependent on labor costs) associated with frontline providers implementing and sustaining a new intervention, opportunity costs need to be estimated since frontline healthcare delivery staff negotiate the tradeoffs with productivity. Providers generally identify patient needs (e.g., preventing falls in an elderly population) as a priority. Yet, when the burden of implementing a new intervention, such as when it takes a substantial portion of their productive time, or when providers do not have access to the support and resources needed to effectively and efficiently implement the EBP, it is unlikely that implementation efforts will be successful.

In contrast, the implementation of policies and practices are usually decided at an upper administration level (organizational level) based on financial incentives. For instance, reducing fall-related injuries is a financial incentive for hospitals so an EBP to address this may be a priority at the policy and/or organizational level [33]. In theory, if fall rates decline, the cost offsets associated with implementation would be realized. However, from the frontline provider’s perspective, this would require more time invested in performing frequent fall-related interventions, which is an opportunity cost for a workforce that must balance competing priorities for their time. For example, an implementation study of a fall-prevention program involved the use of frontline nurses who received additional training and had to support the implementation of the intervention [34]. Administrators at the hospitals wanted to reduce fall rates due to financial incentives based on Medicare policy changes; implementation of the intervention focused on training nursing staff on critical thinking about fall risk and promoting hourly nursing rounds. Implementation of the fall-prevention program yielded net savings between $817,000 and $1,950,000, and the time frontline nurses spent on fall-related activities including fall-risk assessments, assisting with activities of daily living, documenting and ordering fall-related equipment and supplies, and communicating with the medical care team was reduced by 48 to 59%.

This example highlights where a solution (cooperation between the healthcare administrators and frontline nursing staff) resulted in both the hospital administrators and frontline nursing staff benefiting from the fall-prevention program that was implemented at their facilities despite potential conflicts—hospital administrators achieved net savings while frontline staff had more time to devote to other duties.

## Guidelines for including multiple stakeholder perspectives

We provide specific guidelines and recommendations for cost assessment activities involving multiple stakeholders, guided by steps as outlined in Rapid-Cycle Research described by Johnson et al. in Table 2 [35]. Although the guidance in Table 2 would not always produce “rapid” results or actions, the steps are well-suited to issues raised in this paper. For example, in the pre-condition phase, a multi-level stakeholder approach would focus on identifying the stakeholder types including for example the organization, the management team, and the provider team, involved in the implementation effort and impacted by the costs of implementation. We anticipate that this approach in the pre-implementation phase will expand the inclusion of stakeholders and relevant perspectives when considering the costs and consequences of implementation efforts. We apply this approach to multiple stakeholder cost considerations for several reasons. First, this approach is designed to guide systematic and practical approaches to adopting evidence-based practices. Second, it maps on to phases commonly identified across implementation science frameworks (i.e., pre-implementation, implementation, sustainment) and thus can be applied across a variety of implementation efforts. Third, it is designed to guide solution development with the involvement of multiple levels of stakeholders. As this research is still evolving, we also provide hypotheses for future research to confirm, disconfirm, or amend these recommendations.

**Table 2:** Rapid-cycle approach to incorporating multi-level stakeholder economic perspectives when adopting an evidence-based practice. Adapted from Johnson et al. [35]

Phase	Focus	Recommendation	Hypotheses
Preparation (pre-condition)	Identifying multiple stakeholder types	When implementing a new EBP, identify multiple stakeholders across levels who will be impacted by the costs of the implementation.	The inclusion of more stakeholder types initially will set the stage for adopting a multi-level perspective
Problem exploration (pre-implementation)	Understand cost and benefit issues across identified stakeholder levels	Identify costs and priorities across multiple levels of stakeholder groups. Include a rough estimate of costs and discuss with all stakeholder groups involved. Facilitate meetings to discuss cost data and priorities among different stakeholder groups	Such exploration will aid in developing a deeper understanding of costs and priorities across groups and how they influence adoption, implementation, and sustainment
Knowledge exploration (pre-implementation)	Identify areas of cost (and benefit) congruence and conflict across stakeholder levels	Provide key cost information related to priorities of each stakeholder group Investigate organizations that have faced similar issues and have successfully resolved them.	Information summaries using visual displays and emphasizing issues prioritized by that group, should aid in identifying areas where conflicts in priorities need to be resolved
Solution development (pre-implementation)	Identify pragmatic solutions to areas of cost conflict	Facilitate meetings to discuss cost data and priorities, including identifying solutions or reallocation of resources that can be reasonably applied to create win-win scenarios across stakeholder levels	Identifying solutions to resolve incongruencies in stakeholders’ economic priorities will enhance the likelihood of implementation success and EBP sustainment
Solution testing (implementation)	Assess if the solutions worked to reduce incongruencies in cost priorities, resource allocation	Collect (or estimate) costs regularly: early on; mid-implementation; and 6-8 weeks before the program end; include mixed method evaluation to assess effects on costs/priorities of stakeholders.	Collecting costs and other information at multiple points will be worthwhile to make mid-program adjustments in resource allocation if needed and prepare for sustainment and replication
Sustainment	Identifying ongoing costs/benefits, mechanisms for sustainment	Using a multi-level approach, identify resources needed to support implementation efforts, plan for resource allocation, and ongoing cost assessment.	Planning for sustainment from a multi-level cost perspective will enhance the likelihood of institutionalization and efficient use of resources.

As the field of implementation science moves forward with building the evidence base on estimating costs and conducting comparative economic evaluation of implementation, we must specify the perspectives, and cost considerations, including the specific costs relevant to each stakeholder group as a vital first step. There can be considerable variation in costs and cost-effectiveness based on perspective and costing methodology [3], and recognizing these variations and approaches to resolving potential areas of conflict will be essential to advancing the business case for implementation in health care and public health.

### Recommendations

Table 2 summarizes our key steps as identified in this approach. It is important to collect cost data using perspectives across stakeholder groups to successfully plan, implement, sustain, and spread a program. Often costs and priorities of frontline providers or individual participants are not included. As noted in the table, we hypothesize that this will result in inferior long-term results and a lack of sustainment. It is important to not just collect data from these stakeholders but to engage them using participatory approaches [22] and address conflicting economic priorities guided by economic theories. However, theories in these fields were largely developed independently of the social, cultural, and institutional embedding of relationships among stakeholder groups. Future research can build on previous lessons learned by adapting and applying economic theories, as well as related fields such as behavioral economics and decision sciences, for practical application in implementation science. The application of these ideas can aid in understanding areas of congruence and conflict across stakeholder groups and how to successfully support stakeholder cooperation. Theoretically grounded research is critical to understanding key contextual implementation questions such as “what works, when, where, and why” and how to minimize both monetary and non-monetary costs and unintended economic consequences. In addition, applying approaches such as mixed methods (see Solution testing in Table 2 [22];) to these issues will enhance the relevance of implementation economic analyses, and deepen our understanding of the complexities of multiple stakeholder perspectives to more effectively apply economic theory in designing solutions.

Working with stakeholders during pre-implementation phases to “model” approximate costs of an intervention and its associated implementation strategy (or strategy bundles as often used), and to consider their potential impact on resource demands and allocation (as well as benefits) across stakeholder levels, may ultimately save time, money, and enhance cooperation on economic issues. Adapting existing tools, such as the economic impact inventory template posited by Neumann et al., can advance the field by organizing, and presenting various types of costs and consequences from the perspectives in which they occur [2]. Facilitated engagement around implementation cost across types of stakeholders to address these differing perspectives, therefore, may enhance the likelihood of implementation success and sustainment.

### Limitations

This article has several limitations. First, there is not enough literature to conduct a systematic review and there have not, to our knowledge, been comparative studies of different methods to address the issues above. Stakeholders often do not agree and engaging multiple parties in open discussions and problem-solving to come to a consensus is at present more art than science. More research is needed on the specific types and formats of cost feedback to provide to different stakeholders. Cidav et al. [36] provide examples of feedback displays on the costs of implementation strategies, but far more user testing and experimentation are needed in this area. Second, and somewhat ironically, the cost and burden of collecting cost data and providing feedback to multiple parties at multiple points in time can be considerable. This paper is restricted to issues of costs and does not comprehensively consider effectiveness, benefits, or budget impact. Cost is only part of the value equation and by itself is not sufficient to guide decision-making. Issues of conceptualizing, assessing, providing feedback on, and using benefits are covered elsewhere [2, 37] and beyond the scope of this article. Finally, the rapid and frequent adaptations that are often made in implementation projects make costing tricky, as well as attributing costs to specific implementation strategies when ever-changing bundles of strategies are used. These issues are discussed in Quanbeck et al. (Quanbeck A, Cidav Z, Eisman A, Garner B, Glasgow R, Wagner T. Approaches for estimating the cost of implementation strategies, submitted for review) paper included in this special collection.

Other potentially useful theories for advancing multiple stakeholder perspectives in economic evaluation in implementation science, such as those from behavioral economics and decision sciences, were not fully addressed here. Zimmerman et al., for example, have leveraged the concept of bounded rationality as a foundation on which they have built comprehensive simulation models to aid healthcare stakeholders in providing treatment services for veterans with posttraumatic stress disorder [38]. These and other research efforts [12, 39] aim to demonstrate the relevance and practical utility of behavioral economic concepts such as bounded rationality for implementation research and, in particular when considering the decision-making approaches and perspectives of different stakeholders.

## Conclusions and future directions

There has been a recent surge of publications related to the collection of costs in implementation science [9, 14, 31, 36], and emerging consensus regarding the importance of economic influences on implementation outcomes (see other articles in this collection). This paper contributes to economic evaluation by discussing a range of issues involved in considering implementation costs in the real-world from the perspective of multiple stakeholders to advance both the science and practice of implementation. Specifying the key types of stakeholders will aid in putting the deceptively simple advice to “consider multiple perspectives” into practice and inform both researchers and implementers. This paper not only discusses how we can apply economic theory to take steps beyond identifying costs relevant to each stakeholder group but also obtain a deeper understanding of differing perspectives, nonmonetary influences on decision-making, how to recognize (and build upon) areas of overlap, and rectify areas of conflict.

We conclude that there are compelling reasons to collect, report, and understand costs from the perspectives of different stakeholders. Failure to do so may result in (1) lack of program adoption, especially if organizational and policy perspectives are not considered; (2) poor reach to individual participants, especially those at high risk and with few resources; (3) poor quality implementation, especially if not considering the perspective of both supervisory and delivery staff; and (4) poor sustainment when not considering all these perspectives.

Our recommendations are based upon the synthesis of existing literature, input from the diverse disciplines represented on our authorship team, various economic theories, applied experience collecting cost data, and implementation science frameworks, but not on a systematic review or meta-analyses.

The recommendations we offer above and in Table 2 should be tested and revised as necessary based upon their potential usefulness. As with non-economic applications, understanding and working with stakeholders before, during, and after program implementation is essential. We hypothesize that collecting, estimating, reporting, and discussing costs with different types of stakeholders will enhance the adoption, implementation, and sustainment of EBPs. The specific and most appropriate activities, collection strategies, feedback and reporting methods, timing, and frequency of assessments are yet to be determined and will likely vary across settings and contextual factors. Future research should test different methods of cost collection—evaluating their pros and cons and including assessment of the costs and burden of collecting and using cost data by different stakeholders. There is both an important need and a great opportunity to advance implementation science by including and testing different pragmatic approaches to costing from the perspective of different stakeholders.

## Data Availability

Not applicable.

## Glossary

Adaptation: Adaptation is defined as “the degree to which an evidence-based intervention is changed or modified by a user during adoption and implementation to suit the needs of the setting or to improve the fit to local conditions [40, 41].”
Adoption: Adoption is the initial decision or intention of an organization or a community to employ or try an evidence-based intervention [42].
Analytic perspective: Identifies parameters in terms of which costs (inputs) are included in an economic evaluation [43]; the societal perspective includes all costs and benefits, regardless of who pays them or receives the benefits [44].
Decision science: Decision science is the collection of quantitative techniques used to inform decision-making at the individual and population levels. It includes decision analysis, risk analysis, cost-benefit, and cost-effectiveness analysis, constrained optimization, simulation modeling, and behavioral decision theory, as well as parts of operations research, microeconomics, statistical inference, management control, cognitive and social psychology, and computer science. By focusing on decisions as the unit of analysis, decision science provides a unique framework for understanding public health problems, and for improving policies to address those problems [21].
Economic evaluation: The comparative analysis of alternative courses of action in terms of both their costs and consequences [3].
Evidence-based intervention: The focus of D&I activities are on interventions with demonstrated efficacy and effectiveness (i.e., evidence-based). Interventions broadly ca include programs, practices, policies, and guidelines [45].
Implementation strategies: Implementation strategies refer to methods, techniques, activities, and resources to enhance the adoption, integration, and sustainment of evidence-based interventions into clinical or community settings [46, 47].
Implementation costs: Implementation cost is defined as the “cost impact of an implementation effort.” Implementation costs depend on intervention costs, the implementation strategy deployed, and the setting(s) in which the intervention is implemented [42].
Innovation: Innovation refers to a practice, idea or program that is perceived as new by potential adopters. Some use this interchangeably with *evidence-based intervention* [48]*.*
Mixed methods: Mixed methods designs involve the collection and analysis quantitative and qualitative data in a single study to answer research questions using a convergent, sequential or conversion approaches. Mixed methods designs are appropriate to answer complex research questions [49].
Opportunity costs: The value of the next best investment forgone [50].
Sustainability: Sustainability is the extent to which an evidence-based intervention delivers its intended effects over a period of time after external support from the outside agency or funder is finished; three indicators of sustainability include (1) maintenance of program benefits, (2) institutionalization of the program, and (3) capacity building in the recipient organization or community [51]
Sustainment: Activities that build resources and capacity for continued use of an intervention or program within a clinic or community [52].
